# Caesarean Section Delivery Is Associated with Childhood Overweight and Obesity, Low Childbirth Weight and Postnatal Complications: A Cross-Sectional Study

**DOI:** 10.3390/medicina59040664

**Published:** 2023-03-27

**Authors:** Sousana K Papadopoulou, Maria Mentzelou, Eleni Pavlidou, Georgios K Vasios, Maria Spanoudaki, Georgios Antasouras, Anastasia Sampani, Evmorfia Psara, Gavriela Voulgaridou, Gerasimos Tsourouflis, Maria Mantzorou, Constantinos Giaginis

**Affiliations:** 1 Department of Nutritional Sciences and Dietetics, School of Health Sciences, International Hellenic University, 57400 Thessaloniki, Greece; 2 Department of Food Science and Nutrition, School of Environment, University of the Aegean, 81400 Myrina, Greece; 3 First Department of Pathology, Medical School, University of Athens, 11527 Athens, Greece; 4 Second Department of Propedeutic Surgery, Medical School, University of Athens, 11527 Athens, Greece

**Keywords:** caesarean section, childhood obesity, birth weight, postnatal outcomes, pre-school age, vaginal delivery, body mass index, public health, obstetric conditions

## Abstract

*Background and Objectives*: In the last decades, simultaneously increasing trends have been recorded for both caesarean section delivery and childhood overweight/obesity around the world, which are considered serious public health concerns, negatively affecting child health. Aim: The present study aims to investigate whether caesarean section is associated with the increased rates of childhood overweight/obesity, low childbirth anthropometric indices and postnatal complications in pre-school age. *Materials and Methods:* This is a cross-sectional study in which 5215 pre-school children aged 2–5 years old were enrolled from nine different Greek regions after applying specific inclusion and exclusion criteria. Non-adjusted and adjusted statistical analysis was performed to assess the impact of caesarean section in comparison to vaginal delivery. *Results:* Children delivered by caesarean section were significantly more frequently overweight or obese at the age of 2–5 years, also presenting a higher prevalence of low birth weight, length and head circumference. Caesarean section was also associated with higher incidence of asthma and diabetes type I at the age of 2–5 years. In a multivariate analysis, caesarean section increased the risk of childhood overweight/obesity and low childbirth anthropometric indices even if adjusting for several childhood and maternal confounding factors. *Conclusions*: Increasing trends were recorded for both caesarean section delivery and childhood overweight/obesity, which are considered serious public health concerns. Caesarean section independently increased childhood overweight/obesity in pre-school age, highlighting the emergent need to promote health policies and strategies to inform future mothers about its short and long-term risks and that this mode of delivery should preferably be performed only when there are strong medical recommendations in emergency obstetric conditions.

## 1. Introduction

Caesarean section is a crucial medical procedure for saving maternal and infant lives in emergency obstetric circumstances, reducing maternal and neonatal morbidity and mortality [1]. The World Health Organization (WHO) recommends a caesarean section rate of about 10–15% of all live births [2]. However, in the last few decades, there has been a gradual increase in caesarean section deliveries, reaching ‘epidemic proportions’ worldwide [3]. This is a serious public health concern because caesarean sections can negatively affect both maternal and child health even if they can be lifesaving [1,2,3]. 

In Greece, over half of the deliveries occur by caesarean section, making it one of the countries with the greatest caesarean section rates in the world. More pertinent, caesarean section rates increased by almost 50% from 1983 to 1996 [4]. In a previous Greek study, a 41.6% caesarean section rate in two public hospitals and a rate of 53% in a private hospital were reported [5]. The authors of this study supported the view that obstetricians may tend to perform a caesarean section for financial and convenience reasons [6]. In this aspect, the rates of caesarean section deliveries have annually increased more rapidly for women delivering in private health units than those delivering in public hospitals [7].

In its 2015 statement, the WHO reported that caesarean section rates above 10% are not associated with reduced maternal and neonatal mortality and that, ideally, caesarean sections should be performed only when they are medically necessary [8]. Moreover, caesarean section is associated with certain short- and long-term risks, such as a higher prevalence of ectopic pregnancy, fetal death, uterine rupture, premature delivery in subsequent pregnancies or deliveries and a higher incidence of psychiatric symptoms for both the mother and child [9,10]. Short-term risks of caesarean sections on newborns include impaired immune development, higher risk of allergy, atopy and asthma [9], as well as increased respiratory and obesity problems, while long-term health complications include metabolic disorders, diabetes, cardiovascular disease and cancer [11,12,13].

Globally, the number of children affected by obesity has gradually increased tenfold: from 5 and 6 million in 1975 to 50 and 74 million in 2016 for boys and girls, respectively [14]. If these post-2000 trends continue, obesity may affect 25% of all children under 16 years by the end of 2050 [14]. Notably, in the last two decades, the global prevalence of overweight and obesity in children under the age of 5 years old has risen from 32 to 42 million [14]. In this aspect, Greece is considered one of the European countries with a very high rate of childhood obesity and overweight. Notably, the overall prevalence of overweight including obesity is 29.1% in girls, and 31.2% in boys, respectively [15].

Alarmingly enough, once obesity is established in early life, it may extend into adulthood, therefore creating a lifelong condition that is difficult to resolve [16]. Given the current levels of childhood obesity, predictive modelling has suggested that 60% of children today will have obesity by 35 years of age [17]. Obesity is therefore a global public health problem, which is responsible for several adverse consequences. In the short term, children with obesity are at greater risk of adverse physical and psychological comorbidities as well as musculoskeletal difficulties, asthma and obstructive sleep apnea [18]. In the longer term, there is an increased risk of morbidity, including cardiovascular diseases, metabolic disorders, cancer and premature death [19]. The substantial cost of treating the associated health implications has also led to an intense focus on reducing rates of childhood obesity worldwide [20].

Since a similar trend has been observed for both caesarean section delivery and childhood obesity, a possible causal association has been speculated; however, the available studies have currently presented conflicting results. To date, several studies have indicated that caesarean section delivery may be associated with a higher risk of developing childhood and adolescent overweight and obesity compared to vaginal delivery [21,22,23,24,25,26,27,28,29,30]. On the other hand, there are also several studies which have not supported a significant association between caesarean section and childhood or adolescent overweight and obesity [31,32,33,34,35,36,37,38]. It should be noted that some studies did not adjust for potential confounding factors, while others found a moderate or non-association when adjusted for confounders. Thus, the existing results remain inconclusive and the true strength of the association between caesarean section and childhood overweight/obesity remains unclear, let alone when the potential for confounding has not adequately been addressed. The above discrepancies may also be ascribed to the different age groups during childhood or adolescence that were analyzed in the relative studies.

Several studies also indicated that the prevalence of low birth weight was higher in infants delivered by caesarean section than those born with vaginal delivery [39,40,41,42,43,44,45,46,47,48,49]. However, there are also several studies which did not support a significant association between caesarean section and low childbirth weight [50,51], whereas others documented that caesarean section was associated with a lower prevalence for low birth weight [52,53,54]. Thus, there is an urgent need for large scale studies to resolve these controversies. Alarmingly enough, low birth weight has also significant negative consequences for the health and survival of neonates and is an underlying factor in neonatal deaths [55]. Based on the WHO, low birth weight babies are more susceptible to birth asphyxia, trauma, hypothermia, hypoglycemia, respiratory disorders and infections [56]. In addition, low birth weight has long-term consequences in the form of growth inhibition, impairment of cognitive development and increased incidence of chronic diseases such as diabetes type II, hypertension and cardiovascular diseases [57].

Furthermore, there is growing evidence that caesarean section may increase the risk of developing postnatal complications such as diabetes type I and asthma either into childhood or into early adulthood. A substantial meta-analysis on 20 observational studies demonstrated a 20% consistent increase in the risk of childhood diabetes type I after caesarean section [58]. More recent studies also revealed a small but significant and consistent increase in the risk of diabetes type I in children born through caesarean section [59,60,61]. However, other studies did not find any relative association during childhood or adolescence [62,63,64], rendering the overall existing findings controversial and inconclusive. Moreover, several observational studies supported evidence that caesarean section may be one of the risk factors for the development of childhood asthma, although such observations remain inconclusive [65,66,67,68]. Recently, a meta-analysis showed higher risk of developing childhood asthma in the case of caesarean section compared to vaginal delivery [69]. However, the authors speculated that this observation should be interpreted with caution considering the significant heterogeneity of the included studies [69]. In view of all the above considerations, the present cross-sectional study aims to assess whether caesarean section may affect the risk of developing childhood overweight and obesity, childbirth anthropometric indices and certain postnatal complications in pre-school age in a representative children’s population in Greece taking into account multiple confounding factors.

## 2. Methods

### 2.1. Subjects

In the present study, 7035 pre-school children at the age of 2–5 years old and their matched mothers were initially enrolled from 9 geographically diverse Greek regions, namely Athens, Thessaloniki, Larisa, Patra, Alexandroupolis, Kalamata, Ioannina, Crete and North Aegean. Recruitment to the study was between May 2016 and September 2020. The inclusion criteria for the initial enrollment were children aged 2–5 years old whose mothers had a singleton birth before 2–5 years. All participants’ information was confidential, and all participating children were disease-free except from a possible development of asthma or diabetes mellitus I during the postpartum period of 2–5 years. Among the 7035 initially enrolled children and their matched mothers, 962 of them (13.7%) were excluded from the study due to missing or incomplete data. Among the remaining 6073 children and the matched mothers, 858 (14.1%) of the participating children were then excluded from the study due to any history of childhood disease such as neurodevelopment disorders (e.g., autism spectrum disorder, attention deficit hyperactivity disorder, mental retardation, motor disorder), diabetes type II, hypertension, anemia, hyperlipidemia, cancer and autoimmune diseases. The history of the potential disease of the excluded children was reported by their mothers in the given questionnaires. A total of 5215 children and their matched mothers were included in the final analysis after the above inclusion and exclusion criteria, resulting in a final response rate equal to 74.1%. The mothers of the children were informed about the purpose of the study and signed a consent form. Sample size calculation was based on the use of PS: the Power and Sample Size calculator programme, while the randomization was carried with the use of a sequence of random binary numbers (i.e., 001110110 in which 0 represented enrollment and 1 represented not enrollment to the study). A sample size of about 7000 participants was derived by the calculator programme. Hence, from an initial sample of about 12,300 volunteers, 7035 mothers and their paired children were randomly assigned by the use of the above sequence of random binary numbers. The study was approved by the Ethics Committee of the University of the Aegean (ethics approval code: no 12/14.5.2016) and was in compliance with the World Health Organization (52nd WMA General Assembly, Edinburgh, Scotland, 2000).

### 2.2. Study Design

At the time of study, i.e., 2–5 years after delivery, validated semi-quantitative questionnaires were used to assess the sociodemographic, anthropometric and lifestyle characteristics and perinatal and postnatal outcomes of the study population [70,71,72]. The questionnaires were completed by the mothers of the children. The children’s anthropometric measures (weight, length and head circumference) at the time of birth were retrieved by their mothers’ gynecologists’ or hospitals’ medical records. The children’s anthropometric parameters (weight and height) at the age of 2–5 years old (i.e., 2–5 years postpartum) were measured by a trained nutritionist as per protocol. Weight was measured using the same electronic scale, and height was measured using a portable stadiometer [70,71,72]. The International Obesity Task Force (IOTF) reference was used to define overweight and obesity in pre-school children [73,74].

Additionally, the mode of delivery (vaginal or caesarean section) and diabetes mellitus type I was recorded by self-report from the mothers of the study children. Childhood asthma was diagnosed by a specialized physician using questionnaires based on the International Study of Asthma and Allergies in Children and reporting of asthma-specific medication and healthcare use [75]. Mothers were also asked to report if they had a preterm birth (<37th week) and their answers were further cross-checked by their gynecologists’ or hospitals’ medical files for more precise records on the exact week of preterm birth to be obtained; however, we observed that there were several missing data concerning the exact week of preterm birth in the medical records and several of them did not agree with the mothers’ answers and thus preterm birth was treated as the binary outcome as before and after the 37th week of pregnancy. Women’s weight at the first weeks of pregnancy and right before the delivery were retrieved from their personal gynecologists’ or hospitals’ medical files in which measured weight data had been recorded during the mothers visits to healthcare units. The mothers’ pre-pregnancy body mass index (BMI) was calculated from measured weight and height at the first weeks of pregnancy. Gestational weight gain was calculated by subtracting the retrieved measured mothers’ weight at the first weeks of pregnancy from the retrieved measured weight right before the delivery [70,71,72].

Maternal age, nationality, educational and economic status, smoking habits and parity status were retrieved by the mothers’ answers from the questionnaires 2–5 years postpartum and they were based on the mothers’ memory recall. In fact, educational level was scaled according to the sum of years of education and economic status was classified according to the annual family income as: EUR 0 ≤ 5000, EUR 1 ≤ 10,000, EUR ≤ 15,000, EUR 3 ≤ 20,000, EUR 4 ≤ 25,000 and EUR 5 ≥ 30,000. Financial status was further categorized as low for annual family income ≤ EUR 10,000, medium for annual family income ˃ EUR 10,000 and ≤EUR 20,000, and high for annual family income ˃ EUR 20,000 [70,71,72].

The mothers were asked whether they breastfed their children, whether they exclusively breastfed for at least four months and the duration of breastfeeding. To overcome recall bias, the women were asked regarding exclusive breastfeeding for at least four months because at this time point most of them were advised to gradually introduce pulp foods to the feeding practices of their children and they therefore remembered this time point more precisely, rendering their answers more reliable. In contrast, mothers who breastfed for shorter periods were not able to answer the exact duration of breastfeeding with absolute confidence [70,71,72].

Clarifying instructions were given to the mothers of the study children by qualified dietitians and nutritionists regarding the completion of questionnaires while a detailed presentation of the questions to facilitate accurate answers was performed.

### 2.3. Statistical Analysis

Statistical analysis was performed by Student’s t-test for continuous variables followed by finding the normal distribution using the Kolmogorov–Smirnov test. Chi-square test was used for categorical variables. The normally distributed quantitative variables are presented as mean value ± standard deviation (SD) and the qualitative variables as absolute or relative frequencies. Multivariate logistic regression analysis was performed to assess whether mode of delivery is independently associated with childhood anthropometric factors after adjustment for childhood and maternal potential confounders. Differences were considered significant at *p* < 0.05 and 95% Confidence Interval. The statistical analysis of the survey data was performed by Statistica 10.0 software, Europe (Informer Technologies, Inc., Hamburg, Germany).

## 3. Results

### 3.1. Demographic and Anthropometric Characteristics and Perinatal and Postnatal Outcomes of the Study Population

The present study finally enrolled 5215 children aged 2–5 years old and their matched mothers based on the study inclusion and exclusion criteria. The mean age of the children was 4.1 ± 1.1 years (range: 2.0–5.5 years). Concerning the children’s gender, 50.8% of the children were female and 49.2% were male. Regarding BMI classification at the age of 2–5 years, 16.6% of the children were overweight and 7.9% of the children were obese. The mean birth weight of the study population was 3188 ± 513 g (range: 1320–5000 g); 8.3% of them had low newborn weight (<2500 g) and 6.0% of them had high newborn weight (>4000 g). The mean birth length was 46.6 ± 2.0 cm (range: 40–54 cm) and the mean birth head circumference was 36.1 ± 1.9 cm (range: 32–39 cm). As far as the mode of delivery is concerned, 43.5% of them were delivered vaginally and 56.5% of them were born by caesarean section. As far as postnatal outcomes are concerned, 4.5% of study children developed asthma, and 4.3% of them developed diabetes mellitus type I at the age of 2–5 years old.

### 3.2. Mode of Delivery in Association with Demographic and Anthropometric Characteristics of the Participant Children

The female children were significantly associated with higher rates of caesarean section than the male children (Table 1). The children delivered by caesarean section were significantly more frequently overweight or obese at the age of 2–5 years old compared to those delivered vaginally (Table 1). In fact, among the children born by caesarean section, 12.8% were overweight and 3.4% were affected by obesity at the age of 2–5 years old. The incidences of overweight and obesity were increased to 19.5% and 11.4%, respectively, among the children born by vaginal delivery (Table 1). The children delivered by caesarean section had significantly lower birth weight than those born by vaginal delivery (Table 1). Among the children born by vaginal delivery, 3.2% had low newborn weight (<2500 g), while this incidence was increased to 12.3% among the children delivered by caesarean section (Table 1). The children born by caesarean section also had significantly lower rates of birth length and birth head circumference compared to those delivered vaginally (Table 1).

### 3.3. Mode of Delivery in Association with Childhood Postnatal Outcomes

The children born by caesarean section had a significantly higher prevalence of developing asthma at the next 2–5 years of their life compared to those delivered vaginally (Table 1). In fact, among the children born by vaginal delivery, 3.6% developed asthma until the age of 2–5 years old, while this incidence increased to 5.3% among children delivered by caesarean section (Table 1). The children born by caesarean section had a significantly higher prevalence of developing diabetes mellitus type Ι until the age of 2–5 years old compared to those delivered vaginally (Table 1). Among children born by vaginal delivery, 3.6% developed diabetes mellitus type I until the age of 2–5 years old, while this incidence increased to 4.8% among the children delivered by caesarean section (Table 1).

### 3.4. Multivariate Logistic Regression Analysis Assessing the Impact of Caesarean Section Delivery on Childhood Overweight/Obesity after Adjustment for Potential Childhood Confounding Factors

In the multivariate logistic regression analysis, caesarean section delivery was independently associated with childhood overweight/obesity after adjustment for potential childhood confounding factors (Table 2). In fact, the children delivered by caesarean section had a more than two-fold higher risk of developing overweight or obesity at the age of 2–5 years old (Table 2). Caesarean section delivery was also independently associated with childbirth weight and asthma development (Table 2). In particular, the children delivered by caesarean section had a 15% higher probability of being born with low weight and a 26% higher risk of developing asthma at the age of 2–5 years old (Table 2). The children’s age, gender, birth length and head circumference, as well as diabetes type I development did not remain significant in multivariate analysis (Table 2).

### 3.5. Multivariate Logistic Regression Analysis Assessing the Impact of Caesarean Section Delivery on Childhood Overweight/Obesity after Adjustment for Potential Maternal Confounding Factors

In a second multivariate regression model, caesarean section delivery was independently associated with overweight/obesity in children and lower birth weight after adjustment for several maternal risk factors such as maternal age, nationality, pre-pregnancy BMI, educational and economic status, smoking habits, parity, gestational weight gain, preterm birth, gestational diabetes, hypertension and exclusive breastfeeding (Table 3). In fact, the children delivered by caesarean section had an 88% higher risk of developing overweight or obesity at the age of 2–5 years old (Table 3). The children delivered by caesarean section had an 11% higher probability of being born with low weight (Table 3). The older women had a 53% higher risk of delivering by caesarean section (Table 3). The overweight and obese women before pregnancy had a two-fold higher probability of giving birth by caesarean section (Table 3). The mothers presenting excess gestational weight gain and preterm birth showed a 22% and 67% higher risk of giving birth by caesarean section, respectively (Table 3). The mothers giving birth by caesarean section had a 47% higher probability of not exclusively breastfeeding (Table 3).

## 4. Discussion

In the last decades, simultaneously increasing trends have been recorded for both caesarean section delivery and childhood overweight/obesity, and thus a possible causal association has been speculated; however, the available existing studies have presented rather conflicting and inconclusive results. Notably, several studies have supported evidence that caesarean section delivery may increase the risk of developing childhood and adolescent overweight and obesity compared to vaginal delivery [21,22,23,24,25,26,27,28,29,30]. In accordance with the above studies, we found that children delivered by caesarean section had a two-fold higher risk of developing overweight or obesity at the age of 2–5 years in a representative sample of nine geographically diverse Greek regions, even if we adjusted for several confounding factors such as maternal age, pre-pregnancy BMI, gestational weight gain and preterm birth. 

On the other hand, in a recent cross-sectional Greek study, the type of delivery had no impact on the weight status in early adolescent children aged 10–12 years [31]. In this aspect, it should be noted that Pei et al. asserted that the type of delivery could affect body weight development only in early childhood, whereas it had no significant effect at the age of 10 years [76]. In support of this view, a prospective study by Yuan et al. showed a marginally significant positive effect of caesarean section on the prevalence of obesity in childhood, though the significance level seemed to decrease as the age increased [77]. Accordingly, in a longitudinal cohort study in Ireland, children at 6 months of age who were born by caesarean section had a significantly higher BMI, but this association did not persist into future childhood [33]. Vinding et al. also showed a higher BMI at 6 months of age in children born by caesarean section compared to those delivered vaginally, whereas this difference did not remain significant into later childhood at the age of 5 or 13 years [35].

Moreover, there are several studies which did not support a significant association between caesarean section and childhood or adolescent overweight and obesity [31,32,33,34,35,36,37,38]. These controversies may be ascribed to the fact that some studies included children of a higher age, e.g., 8–12 years, or focused on adolescents. Another possible explanation for these discrepancies could be the broader range of the children’s age group (e.g., 2–14 years) included in certain of them.

There is also substantial evidence that the incidence of low birth weight was higher in children born by caesarean section than those born by vaginal delivery [39,40,41,42,43,44,45,46,47,48,49]. In line with the above studies, we found that children delivered by caesarean section had a higher incidence of low birth weight than those delivered vaginally. On the other hand, there are some controversial results around the association between low birth weight and caesarean section. For example, Hailu and Kebede [52] reported that caesarean section may exert a preventive effect on low birth weight which could be explained by the caesarean section rates. In fact, it has been speculated that when the rate is within the WHO recommended range, it will be protective against low birth weight; otherwise, it will be a risk factor [40].

As far as postnatal outcomes are concerned, caesarean section seems to slightly increase the risk of diabetes type I in childhood and adolescent [58,59,60,61]. In line with the previous studies, we found a significant increased prevalence of diabetes type I in children delivered by caesarean. However, this increase was only marginal and non-significant after adjustment for confounding factors. This finding is in accordance with certain studies which showed that this association is not consistent with a causal effect, as several confounding factors may account for the elevated risk [59,60,61]. There is also evidence that caesarean section may be associated with a higher risk of childhood asthma [65,66,67,68]. Accordingly, we found a significant association between caesarean section and increased risk of childhood asthma, even if adjusting for several confounding factors. In this context, a recent meta-analysis also revealed an increased risk of childhood asthma occurrence; however, the authors were concerned about the significant heterogeneity of the results of the included studies which did not permit consistent conclusions to be derived [69].

There are some limitations in our study which should be taken into careful consideration. The use of a questionnaire with questions that depend on the memory of the mothers may lead to recall bias. However, there are studies that have shown that parents are able to remember diseases or occurrences with their children. Moreover, epidemiological studies using questionnaires are low cost and extremely useful for generating information. To overcome such recall bias, childhood asthma was diagnosed by a specialized physician using questionnaires based on the International Study of Asthma and Allergies in Children and reporting of asthma-specific medication and healthcare use [73]. However, it should be noted that asthma diagnosis is considered less accurate in young children due to its clinical instability in early years of life [78].

Another limitation deals with the fact that BMI was used to define childhood and maternal overweight and obesity; however, direct measures of body fat mass and distribution are needed to extend and confirm our findings. Additionally, despite a thorough approach to confounding adjustment, we acknowledge the possibility for unmeasured confounding. Even though we have adjusted for multiple confounders such as maternal age, pre-pregnancy BMI, gestational weight gain and preterm birth, it is still possible that residual confounding may have affected our results, while additional lifestyle or health-related factors, such as physical activity and mental health status, could further be included in the adjusted statistical model. Moreover, the children’s medication such as antibiotic use was not recorded. This should be taken into consideration in future studies since antibiotics used during the first 6 month of life have been associated with childhood overweight/obesity [33].

The study’s sample originated from a proportion of Greek areas and thus the generalizability of the findings is restricted to the entire population of children from Greece at 2–5 years of age. However, the study’s sample was enough large and includes children who live in nine geographically diverse Greek regions, including urban and rural areas and islands, thus its representativeness could be considered high enough at least for Caucasian populations. To date, there are no data concerning potential genetic factors (polymorphisms) or population characteristics for Greeks which could explain the controversial results in comparison with the other studies. On the other hand, no conclusions about causality can be made due to the cross-sectional design of our study. Moreover, we had no data concerning the indications of caesarean section for our study sample, which could provide strong explanations as to why low birth weight children were delivered more often by caesarean section.

## 5. Conclusions

This is a cross-sectional study in a representative children’s population in Greece which supported evidence that caesarean section may increase the risk of developing overweight and obesity in pre-school children at the age of 2–5 years old, independently of multiple confounding factors. Caesarean section may also be associated with low childbirth weight and increased risk of developing postnatal outcomes such as childhood asthma and diabetes type I. Future large-scale, well-designed, randomized clinical trials, which will include a greater number of children, and especially a higher proportion of children with postnatal complications are strongly recommended to confirm the present findings. Emergent health policies and strategies should be promoted to inform future mothers that caesarean section is related to certain short- and long-term risks, including childhood overweight obesity, and that this mode of delivery should ideally be performed only when there are strong medical recommendations in emergency obstetric conditions.

## Figures and Tables

**Table 1:** Associations of mode of delivery with childhood demographic and anthropometric characteristics and perinatal and postnatal outcomes.

Characteristics (*n* = 5215)	Mode of Delivery		
	Vaginal (43.5%)	Caesarean Section (56.5%)	*p*-Value
Age (years ± SD)	4.01 ± 1.04	4.10 ± 1.07	*p* = 0.0041
Gender (*n*, %)			*p* = 0.0007
Male	1178 (51.9)	1388 (47.1)	
Female	1092 (48.1)	1557 (52.9)	
BMI at the age of 2–5 years old (*n*, %)			*p* < 0.0001
Normal weight	1903 (83.8)	2036 (69.1)	
Overweight	290 (12.8)	573 (19.5)	
Obese	77 (3.4)	336 (11.4)	
Birth weight (g ± SD)	3280 ± 458	3116 ± 541	*p* < 0.0001
Birth weight status (*n*, %)			*p* < 0.0001
Low newborn weight (<2500 g)	72 (3.2)	363 (12.3)	
Normal newborn weight (2500–4000 g)	2025 (89.2)	2443 (83.0)	
High newborn weight (>4000 g)	173 (3.3)	139 (4.7)	
Birth length (cm ± SD)	47.7 ± 2.0	45.7 ± 1.6	*p* = 0.0016
Birth head circumference (cm ± SD)	37.1 ± 2.0	35.3 ± 1.4	*p* = 0.0075
Asthma (*n*, %)			*p* = 0.0045
No	2188 (96.4)	2790 (94.7)	
Yes	82 (3.6)	155 (5.3)	
Diabetes type I (*n*, %)			*p* = 0.0265
No	2189 (96.4)	2803 (95.2)	
Yes	81 (3.6)	142 (4.8)	

**Table 2:** Multivariate logistic regression analysis assessing the impact of caesarean section delivery on childhood overweight/obesity after adjustment for potential childhood confounders.

Characteristics	Caesarean Section	
	OR * (95% CI **)	*p*-Value
Age (below/over mean value)	1.21 (0.77–1.82)	*p* = 0.2257
Gender (male/female)	1.06 (0.48–1.71)	*p* = 0.2874
BMI at the age of 2–5 years old (normal/overweight or obese)	2.24 (1.83–2.67)	*p* = 0.0033
Birth weight (below/over mean value)	0.85 (0.45–1.22)	*p* = 0.0128
Birth length (below/over mean value)	1.29 (0.47–2.12)	*p* = 0.6870
Birth head circumference (below/over mean value)	1.10 (0.36–2.01)	*p* = 0.5985
Asthma (no/yes)	1.26 (0.80–1.69)	*p* = 0.0249
Diabetes type I (no/yes)	1.09 (0.52–1.88)	*p* = 0.0856

* OR: Odds Ratio. ** CI: Confidence Interval.

**Table 3:** Multivariate logistic regression analysis assessing the impact of caesarean section delivery on childhood overweight/obesity after adjustment for potential maternal confounders.

Characteristics	Caesarean Section	
	OR * (95% CI **)	*p*-Value
BMI at the age of 2–5 years old (normal/overweight or obese)	1.88 (1.62–2.13)	*p* = 0.0081
Birth weight (below/over mean value)	0.89 (0.51–1.33)	*p* = 0.0306
Maternal age (below/over mean value)	1.47 (1.21–1.80)	*p* = 0.0064
Maternal nationality (Greek/other)	0.98 (0.32–1.69)	*p* = 0.5813
Maternal pre-pregnancy BMI (underweight or normal/overweight or obese)	2.01 (1.76–2.33)	*p* = 0.0057
Maternal education level (below/over mean value)	1.36 (0.81–1.98)	*p* = 0.2012
Family economic status (low or medium/high)	1.25 (0.80–1.79)	*p* = 0.0649
Maternal smoking habits (no/yes)	1.67 (1.24–2.11)	*p* = 0.0882
Maternal parity (nulliparity/multiparity)	1.16 (0.67–1.75)	*p* = 0.1879
Gestational weight gain (below/over mean value)	1.22 (0.86–1.67)	*p* = 0.0184
Preterm birth (no/yes)	1.67 (1.28–2.13)	*p* = 0.0218
Gestational diabetes (no/yes)	1.05 (0.21–2.09)	*p* = 0.8893
Pregnancy induced hypertension (no/yes)	1.14 (0.65–1.89)	*p* = 0.3016
Exclusive breastfeeding (yes/no)	0.53 (0.24–0.88)	*p* = 0.0085

* OR: Odds Ratio. ** CI: Confidence Interval.

## References

[1] Betran A., Torloni M.R., Zhang J.J., Gülmezoglu A.M. (2016). WHO statement on caesarean section rates. BJOG.

[2] Betrán A.P., Ye J., Moller A.B., Zhang J., Gülmezoglu A.M., Torloni M.R. (2016). The increasing trend in caesarean section rates: Global, regional and national estimates: 1990–2014. PLoS ONE.

[3] Betran A.P., Ye J., Moller A.B., Souza J.P., Zhang J. (2021). Trends and projections of caesarean section rates: Global and regional estimates. BMJ Glob. Health.

[4] Skalkidis Y., Petridou E., Papathoma E., Revinthi K., Tong D., Trichopoulos D. (1996). Are operative delivery procedures in Greece socially conditioned?. Int. J. Qual. Health Care.

[5] Mossialos E., Allin S., Karras K., Davaki K. (2005). An investigation of Caesarean sections in three Greek hospitals: The impact of financial incentives and convenience. Eur. J. Public Health.

[6] Giaxi P., Gourounti K., Vivilaki V.G., Lykeridoy K. (2022). Which classification system could empower the understanding of caesarean section rates in Greece? A review of systematic reviews. Eur. J. Midwifery.

[7] Einarsdóttir K., Haggar F., Pereira G., Leonard H., de Klerk N., Stanley F.J., Stock S. (2013). Role of public and private funding in the rising caesarean section rate: A cohort study. BMJ Open.

[8] Visser G.H., Ayres-de-Campos D., Barnea E.R., de Bernis L., Di Renzo G.C., Escobar Vidarte M.F., Lloyd I., Nassa A.H., Nicholson W., Shah P.K. (2018). FIGO position paper: How to stop the caesarean section epidemic. Lancet.

[9] Sandall J., Tribe R.M., Avery L., Mola G., Ha Visser G., Se Homer C., Gibbons D., Kelly N.M., Powell Kennedy H., Kidanto H. (2018). Short-term and long-term effects of caesarean section on the health of women and children. Lancet.

[10] Dekel S., Ein-Dor T., Berman Z., Barsoumian I.S., Agarwal S., Pitman R.K. (2019). Delivery mode is associated with maternal mental health following childbirth. Arch. Womens Ment. Health.

[11] Blustein J., Liu J. (2015). Time to consider the risks of caesarean delivery for long term child health. BMJ.

[12] Visser G.H. (2015). Women are designed to deliver vaginally and not by cesarean section: An obstetrician’s view. Neonatology.

[13] Marcoux S., Soullane S., Lee G.E., Auger N. (2023). Association between caesarean birth and childhood cancer: An age-lagged approach. Acta Paediatr..

[14] Abarca-Gómez L., Abdeen Z.A., Hamid Z.A., Abu-Rmeileh N.M., Acosta-Cazares B., Acuin C., Adams R.J., Aekplakorn W., Afsana K., Aguilar-Salinas C.A. (2017). NCD Risk Factor Collaboration (NCD-RisC). Worldwide trends in body-mass index, underweight, overweight, and obesity from 1975 to 2016: A pooled analysis of 2416 population-based measurement studies in 128·9 million children, adolescents, and adults. Lancet.

[15] Tambalis K.D., Panagiotakos D.B., Psarra G., Sidossis L.S. (2018). Current data in Greek children indicate decreasing trends of obesity in the transition from childhood to adolescence; results from the National Action for Children’s Health (EYZHN) program. J. Prev. Med. Hyg..

[16] Geserick M., Vogel M., Gausche R., Lipek T., Spielau U., Keller E., Pfäffle R., Kiess W., Körner A. (2018). Acceleration of BMI in early childhood and risk of sustained obesity. N. Engl. J. Med..

[17] Ward Z.J., Long M.W., Resch S.C., Giles C.M., Cradock A.L., Gortmaker S.L. (2017). Simulation of growth trajectories of childhood obesity into adulthood. N. Engl. J. Med..

[18] Pulgarón E. (2013). Childhood obesity: A review of increased risk for physical and psychological comorbidities. Clin. Ther..

[19] Prospective Studies Collaboration (2009). Body-mass index and cause-specific mortality in 900 000 adults: Collaborative analyses of 57 prospective studies. Lancet.

[20] Sonntag D., Ali S., Bock F.D. (2016). Lifetime indirect cost of childhood overweight and obesity: A decision analytic model. Obesity.

[21] Sulley I., Saaka M. (2022). Relationship between caesarean section delivery and risk of overweight/obesity among children aged 6–23 months in the Tamale Metropolis of Ghana. J. Nutr. Sci..

[22] Ardic C., Usta O., Omar E., Yıldız C., Memis E. (2021). Caesarean delivery increases the risk of overweight or obesity in 2-year-old children. J. Obstet. Gynaecol..

[23] Sogunle E., Masukume G., Nelson G. (2019). The association between caesarean section delivery and later life obesity in 21–24 year olds in an Urban South African birth cohort. PLoS ONE.

[24] Lavin T., Preen D.B. (2018). Investigating caesarean section birth as a risk factor for childhood overweight. Child. Obes..

[25] Cai M., Loy S.L., Tan K.H., Godfrey K.M., Gluckman P.D., Chong Y.S., Shek L.P., Cheung Y.B., Lek N., Lee Y.S. (2018). Association of elective and emergency cesarean delivery with early childhood overweight at 12 months of age. JAMA Netw. Open.

[26] Kuhle S., Tong O.S., Woolcott C.G. (2015). Association between caesarean section and childhood obesity: A systematic review and meta-analysis. Obes. Rev..

[27] Carrillo-Larco R.M., Miranda J.J., Bernabé-Ortiz A. (2015). Delivery by caesarean section and risk of childhood obesity: Analysis of a Peruvian prospective cohort. Peer J..

[28] Salehi-Abargouei A., Shiranian A., Ehsani S., Surkan P.J., Esmaillzadeh A. (2014). Caesarean delivery is associated with childhood general obesity but not abdominal obesity in Iranian elementary school children. Acta Paediatr..

[29] Darmasseelane K., Hyde M.J., Santhakumaran S., Gale C., Modi N. (2014). Mode of delivery and offspring body mass index, overweight and obesity in adult life: A systematic review and meta-analysis. PLoS ONE.

[30] Huh S.Y., Rifas-Shiman S.L., Zera C.A., Edwards J.W.R., Oken E., Weiss S.T., Gillman M.W. (2012). Delivery by caesarean section and risk of obesity in preschool age children: A prospective cohort study. Arch. Dis. Child..

[31] Kanellopoulou A., Antonogeorgos G., Kokkou S., Notara V., Rojas-Gil A.P., Kornilaki E.N., Lagiou A., Kosti R.I., Panagiotakosm D.B. (2022). Assessing the association between breastfeeding, type of childbirth and family structure with childhood obesity: Results from an observational study among 10–12 years old children. Int. J. Food Sci. Nutr..

[32] Smithers L.G., Mol B.W., Jamieson L., Lynch J.W. (2017). Cesarean birth is not associated with early childhood body mass index. Pediatr. Obes..

[33] Masukume G., McCarthy F.P., Baker P.N., Kenny L.C., Morton S.M., Murray D.M., O’B Hourihane J., Khashan A.S. (2019). Association between caesarean section delivery and obesity in childhood: A longitudinal cohort study in Ireland. BMJ Open.

[34] Vehapoglu A., Goknar N., Turel O., Torun E., Ozgurhan G. (2017). Risk factors for childhood obesity: Do the birth weight, type of delivery, and mother’s overweight have an implication on current weight status?. World J. Pediatr..

[35] Vinding R.K., Sejersen T.S., Chawes B.L., Bønnelykke K., Buhl T., Bisgaard H., Stokholm J. (2017). Cesarean delivery and body mass index at 6 months and into childhood. Pediatrics.

[36] Sutharsan R., Mannan M., Doi S.A., Mamun A.A. (2015). Caesarean delivery and the risk of offspring overweight and obesity over the life course: A systematic review and bias-adjusted meta-analysis. Clin. Obes..

[37] Flemming K., Woolcott C.G., Allen A.C., Veugelers P.J., Kuhle S. (2013). The association between caesarean section and childhood obesity revisited: A cohort study. Arch. Dis. Child..

[38] Li H.T., Zhou Y.B., Liu J.M. (2013). The impact of cesarean section on offspring overweight and obesity: A systematic review and meta-analysis. Int. J. Obes..

[39] Çam H., Harunoğulları M., Polat Y. (2020). A study of low birth weight prevalence and risk factors among newborns in a public-hospital at Kilis, Turkey. Afr. Health Sci..

[40] Taha Z., Ali Hassan A., Wikkeling-Scott L., Papandreou D. (2020). Factors associated with preterm birth and low birth weight in Abu Dhabi, the United Arab Emirates. Int. J. Environ. Res. Public Health.

[41] Moreira A.I.M., Sousa P.R.M., Sarno F. (2018). Low birth weight and its associated factors. Einstein.

[42] Momeni M., Danaei M., Kermani A.J., Bakhshandeh M., Foroodnia S., Mahmoudabadi Z., Amirzadeh R., Safizadeh H. (2017). Prevalence and risk factors of low birth weight in the southeast of Iran. Int. J. Prev. Med..

[43] Chen Y., Wu L., Zhang W., Zou L., Li G., Fan L. (2016). Delivery modes and pregnancy outcomes of low birth weight infants in China. J. Perinatol..

[44] Temerinac D., Chen X.I., Sütterlin M., Kehl S. (2015). Influence of fetal birth weight on caesarean section rate and fetal outcome after induction of labor. In Vivo.

[45] Mendes C.Q., Cacella B.C., Mandetta M.A., Balieiro M.M. (2015). Low birth weight in a municipality in the southeast region of Brazil. Rev. Bras. Enferm..

[46] Temerinac D., Chen X., Sütterlin M., Kehl S. (2014). Influence of fetal birth weight on perinatal outcome in planned vaginal births. Arch. Gynecol. Obstet..

[47] Silva A.A., Lamy-Filho F., Alves M.T., Coimbra L.C., Bettiol H., Barbieri M.A. (2001). Risk factors for low birthweight in north-east Brazil: The role of caesarean section. Paediatr. Perinat. Epidemiol..

[48] Daltveit A.K., Vollset S.E., Skjaerven R., Irgens L.M. (1999). Impact of multiple births and elective deliveries on the trends in low birth weight in Norway, 1967–1995. Am. J. Epidemiol..

[49] Silva A.A., Barbieri M.A., Gomes U.A., Bettiol H. (1998). Trends in low birth weight: A comparison of two birth cohorts separated by a 15-year interval in Ribeirão Preto, Brazil. Bull. World Health Organ..

[50] Horrigan T.J. (2001). Physicians who induce labor for fetal macrosomia do not reduce cesarean delivery rates. J. Perinatol. Off. J. Calif. Perinat. Assoc..

[51] Weeks J.W., Pitman T., Spinnato J.A. (1995). Fetal macrosomia. Does antenatal prediction affect delivery route and birth outcome?. Am. J. Obstet. Gynecol..

[52] Hailu L.D., Kebede D.L. (2018). Determinants of low birth weight among deliveries at a referral hospital in Northern Ethiopia. BioMed. Res. Int.

[53] Deshpande J.D., Phalke D.B., Bangal V.B., Peeyuusha D., Sushen B. (2011). Maternal risk factors for low birth weight neonates: A hospital based case-control study in rural area of western Maharashtra, India. New J. Commun. Med..

[54] Sanchez-Ramos L., Bernstein S., Kaunitz A.M. (2002). Expectant management versus labor induction for suspected fetal macrosomia: A systematic review. Obstet. Gynecol..

[55] Darmstadt G.L., Lawn J.E., Costello A. (2003). Advancing the state of the world’s newborns. Bull. World Health Organ..

[56] World Health Organization (2002). Essential Newborn Care and Breastfeeding: Training Modules. Copenhagen, Denmark. apps.who.int/iris/bitstream/handle/10665/107481/e79227.pdf?sequence=1...y.

[57] World Health Organization (2012). Born Too Soon: The Global Action Report on Preterm Birth. Geneva, Switzerland. https://www.who.int/pmnch/media/news/2012/201204_borntoosoon-report.pdf.

[58] Cardwell C.R., Stene L.C., Joner G., Cinek O., Svensson J., Goldacre M.J., Parslow R.C., Pozzilli P., Brigis G., Stoyanov D. (2008). Caesarean section is associated with an increased risk of childhood-onset type 1 diabetes mellitus: A meta-analysis of observational studies. Diabetologia.

[59] Begum M., Pilkington R., Chittleborough C., Lynch J., Penno M., Smithers L. (2019). Caesarean section and risk of type 1 diabetes: Whole-of-population study. Diabet. Med..

[60] Tanoey J., Gulati A., Patterson C., Becher H. (2019). Risk of type 1 diabetes in the offspring born through elective or non-elective caesarean section in comparison to vaginal delivery: A meta-analysis of observational studies. Curr. Diab. Rep..

[61] Khashan A.S., Kenny L.C., Lundholm C., Kearney P.M., Gong T., Almqvist C. (2014). Mode of obstetrical delivery and type 1 diabetes: A sibling design study. Pediatrics.

[62] Stene L.C., Magnus P., Lie R.T., Sovik O., Joner G., The Norwegian Childhood Diabetes Study Group (2003). No association between pre-eclampsia or caesarean section and incidence of type 1 diabetes among children: A large population-based cohort study. Pediatr. Res..

[63] Dahlquist G.G., Patterson C., Soltesz G. (1999). Perinatal risk factors for childhood type 1 diabetes in Europe. The EURODIAB Substudy 2 Study Group. Diabetes Care.

[64] Bache I., Bock T., Volund A., Buschard K. (1999). Previous maternal abortion, longer gestation, and younger maternal age decrease the risk of type diabetes among male offspring. Diabetes Care.

[65] Al Yassen A.Q., Al-Asadi J.N., Khalaf S.K. (2019). The role of Caesarean section in childhood asthma. Malays. Fam. Physician.

[66] Brix N., Stokholm L., Jonsdottir F., Kristensen K., Scher N.J. (2016). Comparable risk of childhood asthma after vaginal delivery and emergency caesarean section. Dan. Med. J..

[67] Black M., Bhattacharya S., Philip S., Norman J.E., McLernon D.J. (2015). Planned cesarean delivery at term and adverse outcomes in childhood health. JAMA.

[68] Davidson R., Roberts S.E., Wotton C.J., Goldacre M.J. (2010). Influence of maternal and perinatal factors on subsequent hospitalization for asthma in children: Evidence from the Oxford record linkage study. BMC Pulm. Med..

[69] Wypych-Ślusarska A., Niewiadomska E., Oleksiuk K., Krupa-Kotara K., Głogowska-Ligus J., Słowiński J. (2021). Caesarean delivery and risk of childhood asthma development: Meta-analysis. Postep. Dermatol. Alergol..

[70] Koutelidakis A.E., Alexatou O., Kousaiti S., Gkretsi E., Vasios G., Sampani A., Tolia M., Kiortsis D.N., Giaginis C. (2018). Higher adherence to Mediterranean diet prior to pregnancy is associated with decreased risk for deviation from the maternal recommended gestational weight gain. Int. Food Sci. Nutr..

[71] Mantzorou M., Papandreou D., Vasios G.K., Pavlidou E., Antasouras G., Psara E., Taha Z., Poulios P., Giaginis C. (2022). Exclusive breastfeeding for at least four months is associated with a higher prevalence of overweight and obesity in mothers and their children after 2–5 years from delivery. Nutrients.

[72] Papandreou D., Mantzorou M., Tyrovolas S., Pavlidou E., Antasouras G., Psara E., Poulios E., Vasios G.K., Giaginis C. (2022). Pre-pregnancy excess weight association with maternal sociodemographic, anthropometric and lifestyle factors and maternal perinatal outcomes. Nutrients.

[73] (2006). WHO Child Growth Standards: Length/Height-for-Age, Weight-for-Age, Weight-for-Length, Weight-for-Height and Body Mass Index-for-Age: Methods and development. http://www.who.int/childgrowth/standards/en/.

[74] Cole T.J., Bellizzi M.C., Flegal K.M., Dietz W.H. (2000). Establishing a standard definition for child overweight and obesity worldwide: International survey. BMJ.

[75] Rosa M.J., Hartman T.J., Adgent M., Gardner K., Gebretsadik T., Moore P.E., Davi R.L., LeWinn K.Z., Bush N.R., Tylavsky F. (2020). Prenatal polyunsaturated fatty acids and child asthma: Effect modification by maternal asthma and child sex. J. Allergy Clin. Immunol..

[76] Pei Z., Heinrich J., Fuertes E., Flexeder C., Hoffmann B., Lehmann I., Schaaf B., von Berg A., Sibylle Koletzko S., Influences of Lifestyle-Related Factors on the Immune System and the Development of Allergies in Childhood plus Air Pollution and Genetics (LISAplus) Study Group (2014). Cesarean delivery and risk of childhood obesity. J. Pediatr..

[77] Yuan C., Gaskins A.J., Blaine A.I., Zhang C., Gillman M.W., Missmer S.A., Field A.E., Chavarro J.E. (2016). Association between cesarean birth and risk of obesity in offspring in childhood, adolescence, and early adulthood. JAMA Pediatr..

[78] Miyake Y., Tanaka K. (2013). Lack of relationship between birth conditions and allergic disorders in Japanese children aged 3 years. J. Asthma.

